# Supplementary material for: Phagocytosis in Marine Coccolithophore Gephyrocapsa huxleyi: Comparison between Calcified and Non-Calcified Strains
Source: Biology (Basel). 2024 Apr 30;13(5):310. doi: 10.3390/biology13050310 (PMC11117637; doi:10.3390/biology13050310)
Supplement: Supplementary file 1 [file biology-13-00310-s001.zip › biology-2971113-supplementary.pdf]

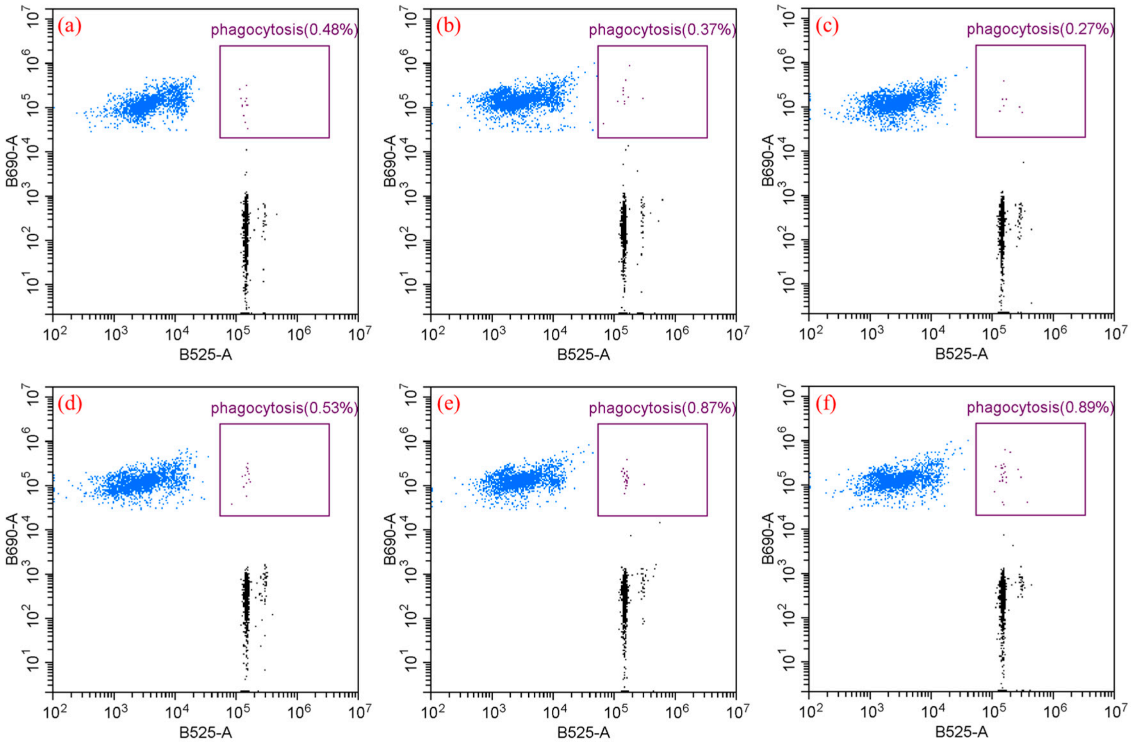

Figure S1 Flow cytometry plots suggesting the phagocytosis percentage in *Gephyrocapsa huxleyi* diploid calcified strain RCC1266 in light (a, b and c, triplicate samples) and darkness (d, e and f, triplicate samples) at 15℃ at the concentration of f/20 medium.

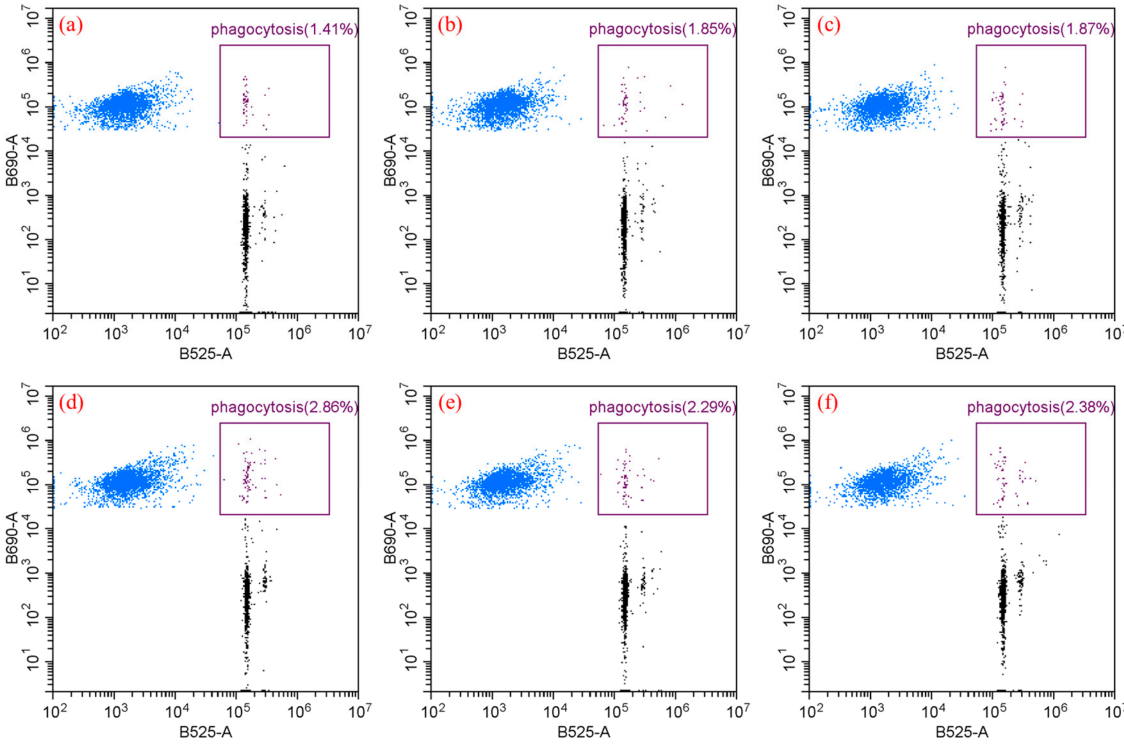

Figure S2 Flow cytometry plots suggesting the phagocytosis percentage in *Gephyrocapsa huxleyi* haploid non-calcified strain PML B92/11 in light (a, b and c, triplicate samples) and darkness (d, e and f, triplicate samples) at

15℃ at the concentration of f/20 medium.

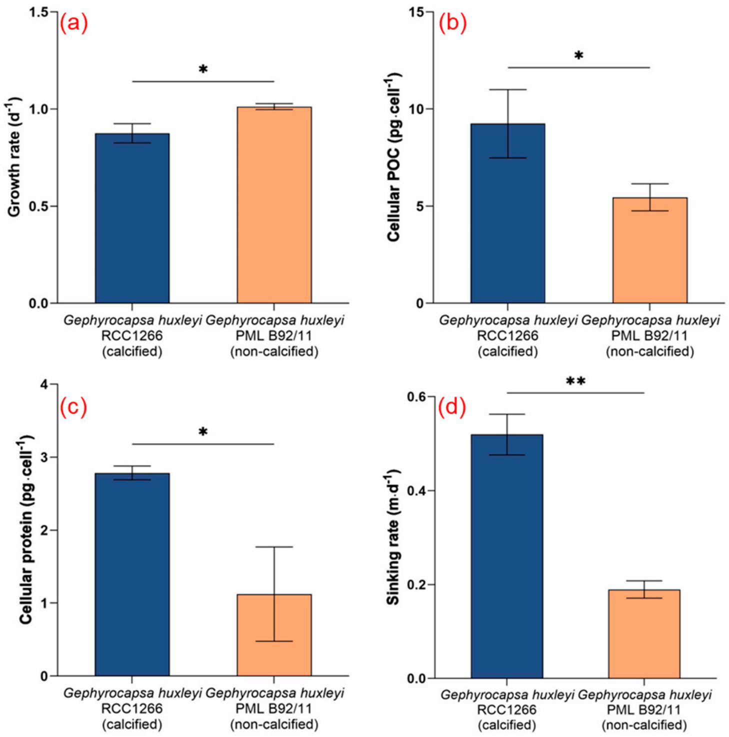

Figure S3 Physiological differences between *Gephyrocapsa huxleyi* diploid calcified strain RCC1266 and haploid non-calcified strain PML B92/11 in growth rate (a), cellular POC (b), cellular protein (c) and sinking rate(d). Replotted from Wang et al. [32]. Significances were determined through t-test. ns: p-value>0.05, *: 0.01<p-value<0.05, **: 0.001<p-value<0.01, ***: p-value<0.001.
